# Genome-Wide Profiling of *Plutella xylostella* Immunity-Related miRNAs after *Isaria fumosorosea* Infection

**DOI:** 10.3389/fphys.2017.01054

**Published:** 2017-12-14

**Authors:** Jin Xu, Xiaoxia Xu, Shuzhong Li, Shuang Wang, Xiaojing Xu, Xianqiang Zhou, Jialin Yu, Xiaoqiang Yu, Muhammad Shakeel, Fengliang Jin

**Affiliations:** ^1^Laboratory of Bio-Pesticide Creation and Application of Guangdong Province, College of Agriculture, South China Agricultural University, Guangzhou, China; ^2^Beijing Genomic Institute, Shenzhen, China; ^3^School of Life Sciences, Central China Normal University, Wuhan, China

**Keywords:** microRNAs, immunity, innate, *Plutella xylostella*, *Isaria fumosorosea*, host pathogen interactions

## Abstract

The development of resistance by *Plutella xylostella* to almost all insecticides is of significant concern all over the world. Entomopathogenic fungi such as *Isaria fumosorosea* have been used as an alternative to insecticides. However, the knowledge of miRNA-regulated reactions against entomopathogenic fungi is still in its infant stage. In the present study, *P. xylostella* was challenged with *I. fumosorosea* at four different time points (12, 18, 24, and 36 h) including a control, to build miRNA libraries by Illumina sequencing. The results of differential expression analysis exhibited that 23 miRNAs were differentially expressed, compared to control, in all treatments. It is worth mentioning, of these, some conserved miRNAs such as miR-2, miR-9a, miR-745, miR-7b, and miR-2767, known to play critical roles in host-pathogen interaction, were also identified. Furthermore, differentially expressed miRNAs were validated by RT-qPCR. Our results provide an essential information for further functional studies of the interaction between *I. fumosorosea* and *P. xylostella* at the post-transcriptional level.

## Introduction

The diamondback moth, *Plutella xylostella* (L.) (Lepidoptera: Plutellidae), is recognized as a major invasive pest of Brassica crops worldwide. The annual control and damage costs for this pest has reached approximately at 4–5 billion dollars globally (Zalucki et al., 2012). The use of chemicals is considered as the major tool for suppressing *P. xylostella* populations, however, this pest quickly evolves insecticide resistance (Shakeel et al., 2017a). The growing concern of insecticide resistance coupled with their harmful effects on the environment has drawn the attention of worldwide researchers toward the development of alternative control strategies (Shakeel et al., 2017a). Therefore, the use of biological control agents, such as entomopathogenic fungi, has received an increased attention. There are several benefits of employing fungal biological control agents, including a decreased impact on the environment, less chance of resistance development, and decreased no-target effects (Lai and Su, 2011; Fan et al., 2012; Smalling et al., 2013). A number of entomopathogenic fungi have been isolated and used to control several insect pests, including *P. xylostella* (Altre et al., 1999; Leemon and Jonsson, 2008; Bukhari et al., 2011). Amongst them, *Isaria fumosorosea* has received attention to be used as a potential fungal biological control agent and has been used in various mycopesticides worldwide (Zimmermann, 2008).

The field of immunology, one of the fascinating facets of biology, has always attracted researchers to elucidate the mechanisms, molecular and cellular, involved in sensing and neutralizing the infectious foreign agents (Imler, 2014). All multicellular organisms have developed a potent and diversified immune system to protect themselves from infectious microorganisms. Insects represent by far the most numerous and diverse group of multicellular organisms. Although insects lack adaptive immunity, specialized defense system of vertebrates, they do have innate immunity that is consisted of cellular and humoral immune responses (Hultmark, 1993). The cellular innate immune response is mainly mediated by hemocytes and comprises phagocytosis, encapsulation, and nodulation (Lavine and Strand, 2002). The insect humoral reactions involve clotting, melanization, and production of potent antimicrobial peptides (Hoffmann and Reichhart, 2002).

MicroRNAs, small non-coding RNA molecules of 18–24 nucleotides in length, are vital regulators of gene expression at the post-transcriptional level in metazoans (Nehammer et al., 2015). In eukaryotes, gene expression is regulated by miRNAs via specific base-pairing with the 3′ untranslated regions (UTRs) of corresponding target genes (Bartel, 2009). There is an increasing number of reports that miRNAs play vital roles in many physiological processes, including development, apoptosis, cell division and differentiation, and immune challenge (Brennecke et al., 2003; Stark et al., 2003; Leaman et al., 2005; Asgari, 2011). While there is a well-established information available about the role of miRNAs in vertebrate development, knowledge is limited about their roles in insect host-pathogen interactions (Hussain and Asgari, 2014). Although the role of insect miRNAs against viruses is recognized, there is no report, until now, according to our information, on miRNA-regulated reactions against entomopathogenic fungi such as *I. fumosorosea*.

Previously, our results of RNA-Seq and differentially expressed gene expression (DGE) analysis of destruxin A and *I. fumosorosea* treated *P. xylostella* exhibited that most of the immunity-related genes were up-regulated in response to destruxin A injection, whereas *I. fumosorosea* has the ability to suppress the immune system of *P. xylostella* (Shakeel et al., 2017c; Xu et al., 2017). Therefore, given the fact that miRNAs play important role in host-pathogen interaction, herein, we aimed to explore the response of *P. xylostella* miRNAs to *I. fumosorosea*, and to determine how the abundance of differential expression of known and novel miRNAs changes following an infection and whether it varies at different times of infection. To achieve these results, we profiled miRNA expression in *P. xylostella* infected with *I. fumosorosea* at 12, 18, 24, and 36 h time points with a control using small RNA deep sequencing.

## Materials and methods

### Insect stock

The susceptible population of *P. xylostella* was maintained under insecticide free conditions for 10 generations in the Engineering Research Centre of Biological Control, Ministry of Education, South China Agricultural University (SCAU). The insects were kept at 60–70% relative humidity and at 25 ± 1°C under a 16:8 h light: dark cycle.

### Fungal strain and samples collection

Stain IfB01 of *I. fumosorosea* (China Center for Type Culture Collection access number: CCTCC M 2012400) was grown on potato dextrose agar (PDA) at 26°C. The conidia were prepared as described previously (Huang et al., 2010). Healthy third instar larvae of *P. xylostella* were selected and treated with 1 × 10^7^ spores/ml suspension and then surviving larvae (50) were collected at 12, 18, 24, and 36 h, post-treatment. The control group larvae were treated with sterile deionized water containing 0.05% Tween-80 and the samples were collected at 0 h post-treatment.

### RNA extraction, small RNA library construction, and sequencing

Trizol Total RNA Isolation Kit (Takara, Japan) was used to extract total RNA from normal and treated larval samples following manufacturer's instructions. The concentrations of RNA were assessed using Nanodrop (Bio-Rad, USA) and its integrity was determined on Agilent 2100 Bioanalyzer (Agilent, USA). The small RNA libraries were constructed from each time-point of infection using a TruSeq small RNA sample preparation kit (Illumina). Briefly, RNAs were firstly ligated with 3′ adapter and after size fraction ligated to 5′ adapter. The small RNA fractions were then used for reverse transcription following PCR. The final ligation PCR products, after purification, were sequenced using Illumina Genome Analyzer (San Diego, CA, USA) at the Beijing Genomics Institute (BGI, Shenzhen, China).

### Bioinformatics analysis of small RNA sequences

To screen clean reads, raw data reads were filtered to remove low-quality, 5′ primer contaminants, without 3′ primers and insert tag, and sequences fewer than 18 nucleotides. The remaining high-quality reads were initially mapped *to P. xylostella* genome (GCA_000330985.1) using Bowtie software (Langmead and Salzberg, 2012), and then annotated into different classes to remove rRNA, scRNA, snoRNA, snRNA, and tRNA using Rfam database. Finally, the unannotated clean sequences were used to predict novel miRNAs using the miRDeep2 software.

#### Differential expression analysis of miRNAs

The expression of miRNAs was compared between treatment and control to identify differentially expressed miRNAs. First, the expression of miRNA in the five libraries was normalized to transcripts per million (TPM). If the normalized expression of the miRNA was 0, it was modified to 0.01 to enable calculation. If the normalized expression of the miRNA was less than 1 in all libraries, it was ignored to compare for low expression. The normalization formula was:

$$\begin{array}{l} \text{Normalized expression}=\text{Actual miRNA count}\\ \;/\text{Total count of clean reads}\times 10^{6}. \end{array}$$

The normalized data were then used to calculate fold-change values and *P*-values, and a scatter plot of the fold-change values was generated. Fold-change was calculated as;

Fold-change = log_2_ (Treatment/Control).

The *P*-value was calculated by the following equation:

$$\begin{array}{l} p\left(x|y\right)={\left(\frac{N_{2}}{N_{1}}\right)}^{y}\frac{\left(x+y\right)!}{x!y!{\left(1+\frac{N_{2}}{N_{1}}\right)}^{\left(x+y+1\right)}}\begin{array}{c} C\left(y\leq y_{\min}|x\right)=\overset{y\leq y_{\min}}{\underset{y=0}{\sum }}p\left(y|x\right)\\ D\left(y\geq y_{\max}|x\right)=\overset{\infty }{\underset{y\geq y_{\max}}{\sum }}p\left(y|x\right) \end{array} \end{array}$$

Where *x* represents small RNA total clean reads in the control, *y* represents total clean reads in the treatment, *N*_1_ represents the normalized expression of a miRNA in library control, and *N*_2_ represents the normalized expression of the same miRNA in library treatment. The corrected *P*-value corresponds to differential gene expression test using Bonferroni method (Abdi, 2007).

### miRNA target prediction and functional analysis

The potential mRNA targets of differentially expressed miRNAs were predicted and analyzed using three different programs, such as RNAhybrid, miRanda, and TargetScan following already established criteria for target prediction (Allen et al., 2005; Schwab et al., 2005). To get more reliable results, we selected those mRNA targets which were predicted by all three programs. Additionally, functional annotation of all the predicted target genes was conducted by using Gene Ontology (GO) database and Kyoto Encyclopedia of Genes and Genomes (KEGG) pathway analyses, with the threshold set at a corrected *P*-value ≤ 0.05.

### RT-qPCR validation

Real-time quantitative PCR (RT-qPCR) is the method of choice for analyzing expression of genes and to confirm the results of RNA-Sequencing (Shakeel et al., 2017b). Thus, to confirm the results of sRNA-Seq in the current study, RT-qPCR analysis was conducted to ensure the expression levels of miRNAs displayed by Illumina sequencing results and 10 miRNAs were selected. RT-qPCR was performed on a Bio-Rad iQ2 optical system (Bio-Rad) using SsoFast EvaGreen Supermix (Bio-Rad, Hercules, CA, USA) following the instructions of the manufacturer. The U6 snRNA was used as an internal control. The reaction program was set as 95°C for 30 s, 40 cycles of 95°C for 5 s, and 55°C for 10 s with a dissociation curve generated from 65 to 95°C to ensure the purity of PCR products (Shakeel et al., 2015). Each experiment was replicated in triplicate. Finally, data analysis was performed using *2*^−Δ*ΔCT*^ method (Livak and Schmittgen, 2001).

## Results and discussion

### Overview of small RNA dataset

To identify miRNAs in *I. fumosorosea* challenged *P. xylostella*, we constructed five small RNA libraries (Tween (TW), 12, 18, 24, and 36 h) using high-throughput Illumina sequencing platform. In total, 11,861,547; 11,872,699; 11,944,980; 11,956,814, and 11,866,077 raw reads were obtained, respectively. After low-quality sequences, adaptors, and sequences less than 18 nucleotides were discarded, 92.93, 98.17, 98.96, 98.63, and 94.62% clean reads were obtained in TW, 12, 18, 24, and 36 h, respectively, for further analysis (Table 1).

**Table 1:** The classification of total small RNAs of the *Plutella xylostella* by sequencing.

**Type**	**Tween (TW)**		**12 h**		**18 h**		**24 h**		**36 h**	
	**Counts**	**Percent**	**Counts**	**Percent**	**Counts**	**Percent**	**Counts**	**Percent**	**Counts**	**Percent**
High-quality reads	11,861,547	100	11,872,699	100	11,944,980	100	11,956,814	100	11,866,077	100
3′ adapter-null	27,731	0.23	45,757	0.39	3,422	0.03	2,394	0.02	29,205	0.25
Insert-null	8,951	0.08	4,048	0.03	6,282	0.05	4,351	0.04	10,499	0.09
5′ adapter-contaminants	205,353	1.73	61,614	0.52	49,186	0.41	25,940	0.22	75,571	0.64
Smaller than 18 nt	595,179	5.02	105,692	0.89	65,511	0.55	131,053	1.1	522,744	4.41
PolyA	907	0.01	142	0	130	0	134	0	275	0
Clean reads	11,023,426	92.93	11,655,446	98.17	11,820,449	98.96	11,792,942	98.63	11,227,783	94.62

**Figure 1:**
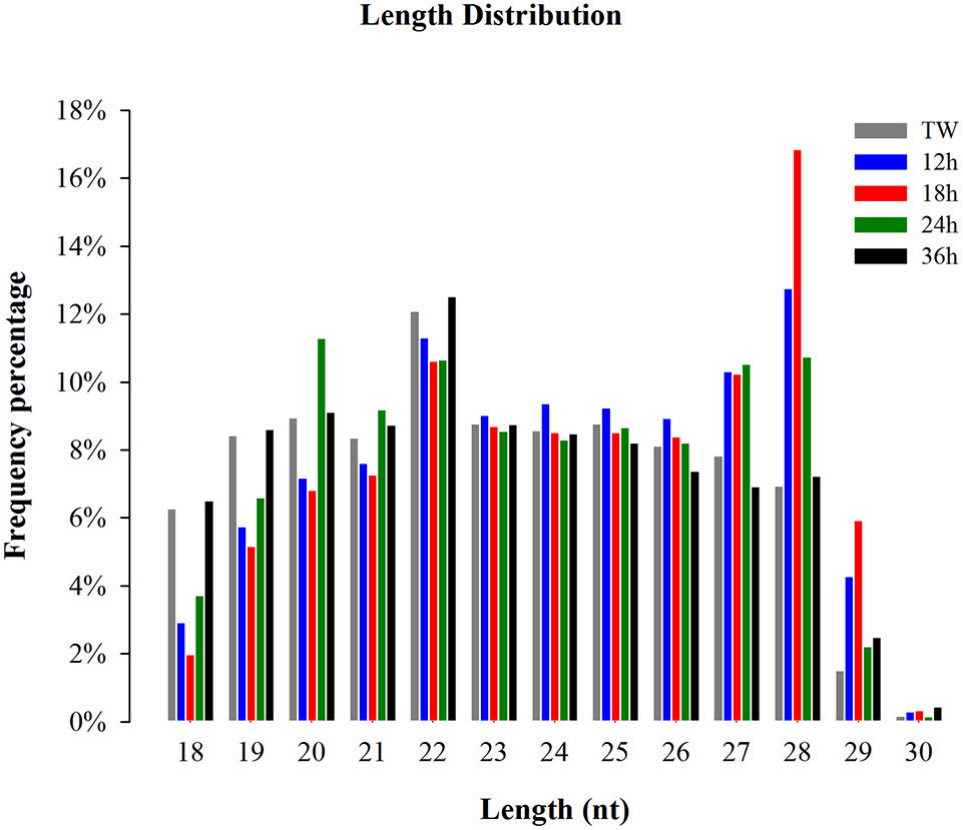
Size distribution of small RNA reads in the libraries of *Plutella xylostella*. Different colors represent different libraries. X-axis represents small RNA length distribution and Y-axis represents frequency percentage. Tween (TW) was used as a control.

The small RNA size distribution in the five libraries showed that most of the sRNAs ranged from 18 to 30 nt, with 28 nt being the most abundant following 22, and 23 nt (Figure 1) in the five libraries. The two peaks observed at 22 and 28 nt, in the present study, represent a typical length of miRNAs and piwi-interacting RNAs, respectively. Our findings are in consistency with the typical size of miRNAs and piwi-interacting RNAs in previous reports (Wei et al., 2009; Etebari et al., 2013; Xu et al., 2015; Li et al., 2016). Among the clean reads, 85.10% sRNAs were common between 12 and 18 h, 85.31% sRNAs between 24 and 12 h, 83.28% sRNAs between 24 and 18 h, 83.92% sRNAs between 24 and 36 h, 84.55% sRNAs between 24 h and TW, 84.89% sRNAs between 36 and 12 h, 83.91% sRNAs between 36 and 18 h, 86.44% sRNAs between 36 h and TW, 85.77% sRNAs between TW and 12 h, and 84.18% sRNAs between TW and 18 h, respectively (Supplementary Figure 1).

### Genome mapping and small RNA annotation

Of the clean reads, 6,784,087, 6,965,570, 7,410,628, 7,023,256, and 6,510,880 reads from control, 12, 18, 24, and 36 h accounted for 61.54, 59.76, 62.69, 59.55, and 57.99%, respectively, and were mapped to the genome of *P. xylostella* (Supplementary Table 1). The annotation of sRNAs was carried out by following priority rule of rRNA etc.; (GenBank>Rfam) > known miRNA > repeat > exon > intron (Calabrese et al., 2007). The clean reads were categorized into miRNA, rRNA, snRNA, snoRNA, tRNA, and unannotated (unann). The composition of the sRNA classes in each library is displayed in Supplementary Figure 2.

### Identification of known and novel miRNAs

After successful mapping of clean reads against *P. xylostella* genome, the mapped miRNA sequences were matched to miRNAs reported by Etebari and Asgari (2016). Our analysis initially identified, based on sequence similarity, in total, 191 mature miRNAs. Then, precursor sequences of these mature miRNAs were aligned to those reported by Etebari and Asgari (2016), and 102 highly confident precursor miRNAs, which produced 172 of 194 mature miRNAs. Our analysis indicated that precursor miRNA sequences of the remaining 22 conserved miRNAs were not detectable in the current assembly of *P. xylostella* genome. After removing those known miRNAs with read count < 10 in all libraries, remaining 116 known miRNAs with precursor sequences (Supplementary Table 2), and 15 miRNAs without precursor sequences (Supplementary Table 3) were retained for further analysis. The remaining sequences that were not matched to conserved miRNAs were used to predict novel miRNAs by using miRDeep2 program (Friedländer et al., 2012). The prediction of novel miRNAs analysis predicted 42 potential novel miRNAs from all the libraries (Supplementary Table 4) following the standard criteria of novel miRNA prediction with a miRDeep score >1, randfold *P*-value < 0.05, and MFE < −19 kcal/mol.

It is worth mentioning that a low copy number of miR-1, a conserved miRNA, was detected after parasitization in a previous report (Etebari et al., 2013), however, in the present study, miR-1 was the most abundant miRNA following pxy-let-7-5p, pxy-miR-184-3p, pxy-miR-10-3p, and miR-31-5p (Table 2). The abundant and common expression of these conserved miRNAs indicates that these miRNAs might play crucial roles in *P. xylostella*. Our results are in consistency with previous reports where a high expression of these miRNAs was observed in other insect small RNA libraries (Cai et al., 2010; Cristino et al., 2011; Liu et al., 2012). Bantam, a most abundantly expressed miRNA, plays multiple roles in insects such as apoptosis inhibition, cell proliferation and stem cell stem cell maintenance, and immunity in *Drosophila melanogaster* (Smibert and Lai, 2010; Fullaondo and Lee, 2012). Although a high copy number of bantam was observed in our study, however, its up-regulation after infection was less than 1-fold. Let-7, a highly conserved miRNA, has also been reported to play an important role in immunity, for example, it binds to 3' UTR of antimicrobial peptide diptericin to repress translation of this protein in *D. melanogaster* (Garbuzov and Tatar, 2010). Interestingly, we found that few miRNAs like miR-2755, miR-10, and miR-31 showed high expression in all treatments. A higher expression of these miRNAs after fungal treatment indicates that these miRNAs might play important roles in defending *P. xylostella* against pathogens.

**Table 2:** Top 10 most abundant miRNAs commonly expressed in the five libraries of *Plutella xylostella*.

**miR_name**	**Mature sequence**		**Counts**			
		**Tween (TW)**	**12 h**	**18 h**	**24 h**	**36 h**
pxy-mir-1-3p	TGGAATGTAAAGAAGTATGGAG	371,221	289,079	276,684	241,868	404,656
pxy-let7-5p	TGAGGTAGTAGGTTGTATAG	77,144	62,823	67,701	101,438	64,418
pxy-mir-184-3p	TGGACGGAGAACTGATAAGGGC	45,689	37,401	40,854	48,942	27,575
pxy-mir-10-3p	CAAATTCGGTTCTAGAGAGGTTT	18,052	11,877	12,032	13,447	16,493
pxy-mir-31-5p	AGGCAAGATGTCGGCATAGCTGA	12,857	11,904	13,039	12,037	10,224
pxy-mir-2755-3p	CACCCTGTCAGACCATACTTGTT	11,483	10,586	10,295	13,527	8,105
pxy-miR-281-5p	AAGAGAGCTATCCGTCGACAGT	9,156	10,361	10,020	7,132	10,957
pxy-mir-10-5p	TACCCTGTAGATCCGAATTTGT	6,647	4,482	4,503	6,458	5,884
pxy-mir-276-3p	TAGGAACTTCATACCGTGCTCT	4,6 99	3,004	2,867	2,217	6,354
pxy-mir-279c-3p	TGACTAGATCCATACTCGTCTG	4,658	5,833	5,468	7,341	6,376

The novel miRNA analysis identified 42 potential novel miRNAs in *P. xylostella* after infection (Supplementary Table 4). Among novel miRNAs, pxy-novel-miR-26 was the abundantly expressed miRNA following pxy-novel-miR-1, pxy-novel-miR-33, and pxy-novel-miR-35 (Supplementary Table 4).

### *I. fumosorosea* responsive miRNAs

**Figure 2:**
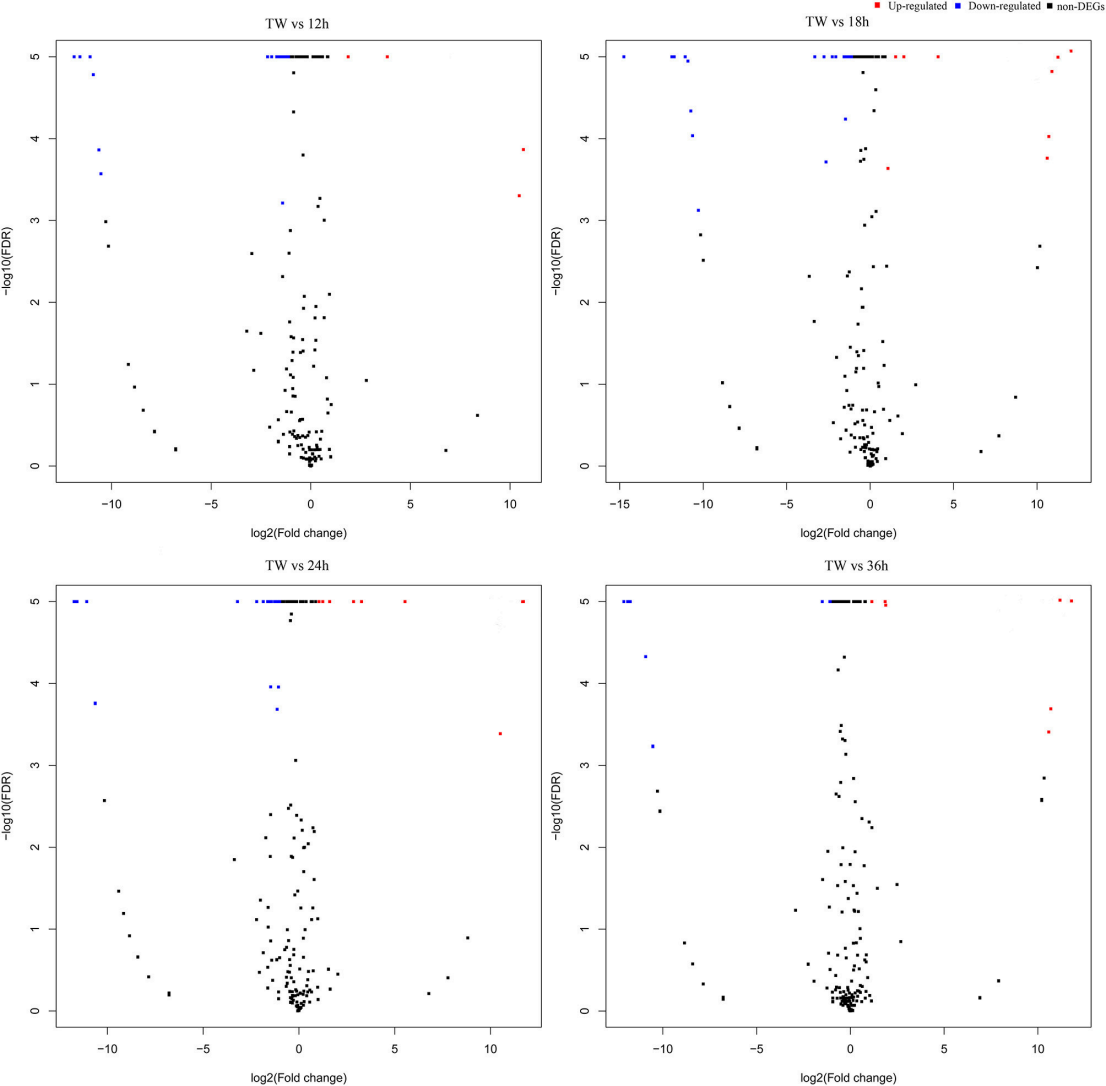
Volcano plot of differentially expressed microRNAs in *Plutella xylostella* post-infection. The volcano plots represent differentially expressed miRNAs at different time points (12, 18, 24, and 36 h) post-infection compared to control.

The differential abundance of host miRNAs, a common observation in host-pathogen systems, changes at different infection stages following an infection (Asgari, 2011). In the present study, to find out the *I. fumosorosea* responsive miRNAs, a differential expression analysis was performed using the sequencing results (Figure 2). The differential expression analysis exhibited that 13, 12, 16, and 5 known miRNAs were differentially expressed in 12, 18, 24, and 36 h, respectively, compared to control (Supplementary Table 5). The top five differentially expressed known miRNAs are presented in Table 3. Furthermore, 12, 19, 13, and 11 novel miRNAs were differentially expressed, in 12, 18, 24, and 36 h, respectively, compared to control (Supplementary Table 6).

**Table 3:** Five common differentially expressed miRNAs at 12 and 18 h compared to Tween (TW) in *Plutella xylostella*.

**miRNA**	**TW (TPM)**	**12 h (TPM)**	**12 h/TW**	***P*-value**	**FDR**	**18 h (TPM)**	**18 h/TW**	***P*-value**	**FDR**
pxy-mir-7b-5p	17.64	3.92	−2.169925	3.29E-20	2.44E-19	2.6	−2.762267033	5.81E-27	5.52E-26
pxy-mir-2768-3p	14.23	4.35	−1.709848	1.94E-12	9.50E-12	5.1	−1.48036651	1.35E-10	6.30E-10
pxy-mir-79-3p	26.74	8.92	−1.583884	5.50E-20	3.82E-19	9.06	−1.56141651	4.42E-20	3.36E-19
pxy-mir-8507-3p	124.38	43.84	−1.504435	8.98E-81	1.61E-79	57.56	−1.111616025	2.44E-52	3.97E-51
pxy-mir-2a-3p	11.49	5.11	−1.168984	2.08E-06	6.78E-06	2.39	−2.265296275	1.16E-14	6.63E-14

Interestingly, in the present study, we found that the expression of few conserved miRNAs like miR-2, miR-9, miR-279, miR-745, miR-7b, and miR-2767 was changed following the infection of *I. fumosorosea*. Our findings suggest that these miRNAs might play very important roles in *P. xylostella* immunity to *I. fumosorosea*. In accordance to our study, previous reports also suggested the important roles of these conserved miRNAs in the immunity of different insects against different pathogens, such as bacteria-injected larvae of *Manduca sexta* and *Diadegma semiclausum* parasitized *P. xylostella* resulted in differential expression of miR-2, miR-9, and miR-279, indicating the role of these miRNAs in immunity against bacteria and parasite, respectively (Zhang et al., 2012; Etebari et al., 2013). It is of note that miR-9 has been predicted to play an essential role in signal recognition in *M. sexta*, and in toll pathway in *Drosophila melanogaster* (Fullaondo and Lee, 2012). It is worth mentioning that the read number of most of the miRNAs dropped after infection and, overall, only 3 miRNAs (miR-282,−2796, and −34) were up-regulated while 20 miRNAs were down-regulated in all the treatments compared to control. Previously, it has been reported that when *Galleria mellonella* was infected with entomopathogenic fungi, *Metarhizium anisopliae*, at larval stage, only one miRNA (miR-210b) showed differential expression (Mukherjee and Vilcinskas, 2014), whereas, in our study, 23 miRNAs were differentially expressed, however, miR-210b was not detected in our small RNA libraries.

### Validation of differentially expressed miRNAs by RT-qPCR

**Figure 3:**
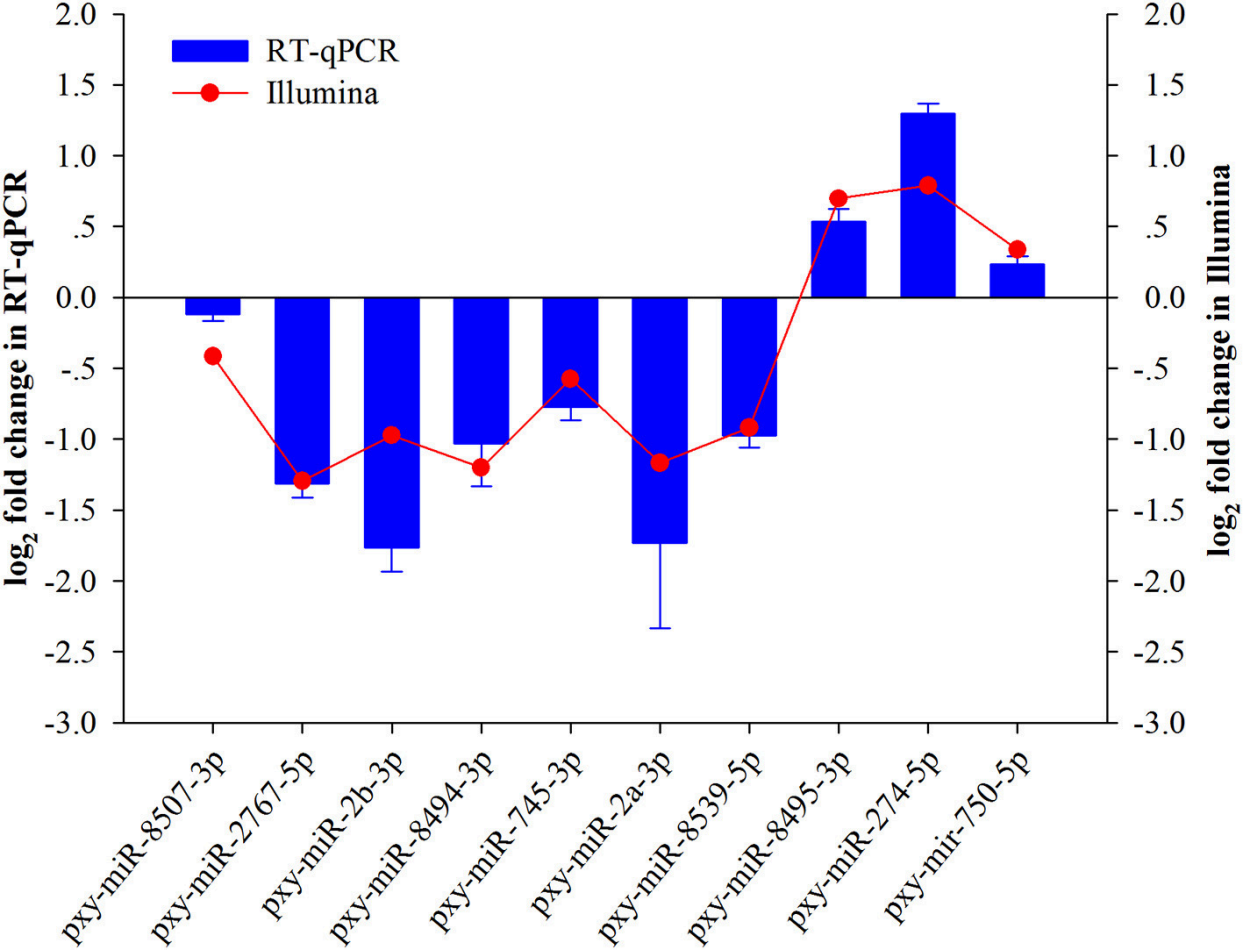
Validation of expression of ten miRNAs achieved by RT-qPCR and sRNA-Seq in *Plutella xylostella* after *Isaria. fumosorosea* infection. Error bars represent ± SD from three independent experiments. U6 snRNA was used as an internal control.

To validate small RNA sequencing results, 10 randomly selected miRNAs were analyzed by RT-qPCR (Figure 3). The results exhibited that the trend of the expression level of the selected miRNAs showed consistency with sequencing results except for a few miRNAs like pxy-miR-2a-3p, pxy-miR-2b-3p, and pxy-miR-274-5p.

### Prediction and annotation of miRNA target genes

**Figure 4:**
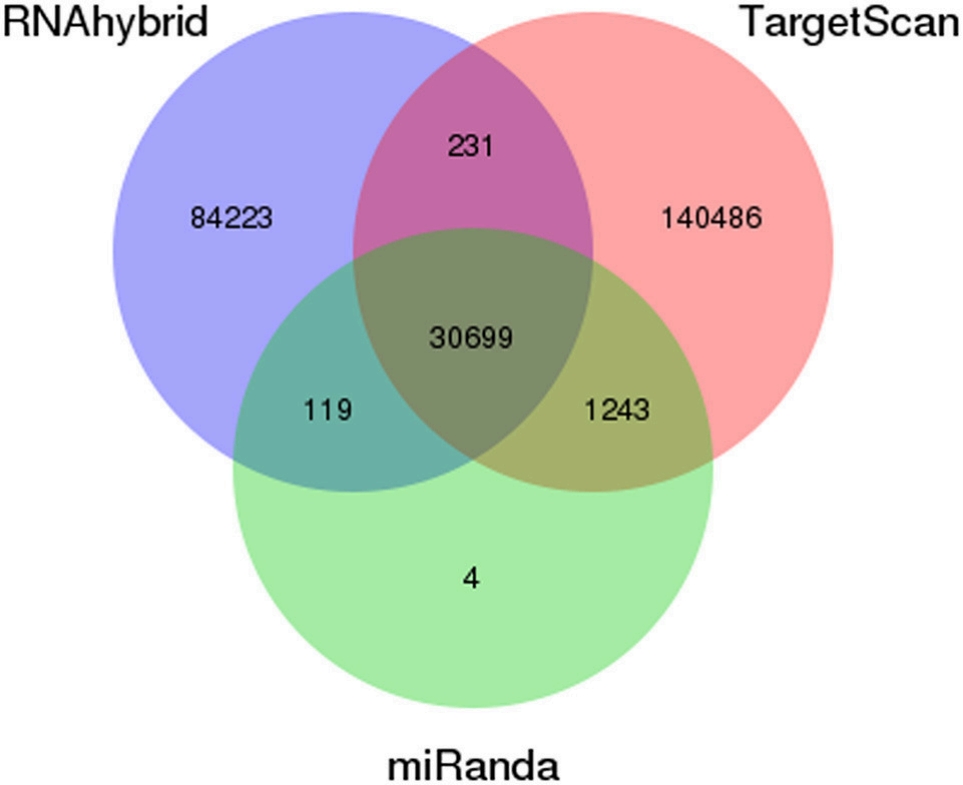
Prediction of potential target genes in all libraries. Venn diagram show the number of miRNA targets and their overlapping spots predicted by the three programs (RNAhybrid, miRanda, and TargetScan).

To better understand the function of differentially expressed miRNAs, putative target genes were predicted using the genome of *P. xylostella* using RNAhybrid, miRanda, and TargetScan software. Our target prediction results indicated that 30,930 common spots were detected between RNAhybrid and TargetScan, 30,818 between RNAhybrid and miRanda, and 31,942 between TargetScan and miRanda. When the target prediction results of all three software were combined, 30,699 common spots were detected and were selected for further analysis (Figure 4).

### Gene ontology and kyoto encyclopedia of genes and genomes analysis

The GO enrichment analysis was performed to classify the functions of miRNA target genes. The putative target genes were classified into three major categories, biological process, cellular component, and molecular function, of GO classification. Our results exhibited that cellular process, cell part, and catalytic activity were the most enriched categories in the biological process, cellular component, and molecular function, respectively, at all-time points of treatment (Supplementary Figure 3). Similar to our findings, previously, target genes of *Ostrinia. furnacallis* in response to *Bacillus thuringiensis* and *Wolbachia*- responsive miRNAs in *Tetranychus urticae* were also categorized into the cellular process, cell part, and catalytic activity (Rong et al., 2014; Xu et al., 2015).

To find out particular signaling pathways of the putative miRNA target genes, Kyoto Encyclopedia of Genes and Genomes (KEGG) analysis was performed. The most enriched categories were transport and catabolism, signal transduction, and cancers in cellular processes, environmental information processing, and human diseases, respectively, at all-time points of infection (Supplementary Figure 4).

## Conclusion

In conclusion, using high-throughput sRNA sequencing, we screened out *I. fumosorosea* responsive immunity-related miRNAs in *P. xylostella*. Based on our knowledge, this is the first study about immunity-related miRNA profiles of *P. xylostella* in response to *I. fumosorosea*. The major finding of this study is the identification of conserved immunity-related differentially expressed miRNAs such as miR-2, miR-9, miR-92, miR-745, and miR-2767. Our findings provide an essential information for further functional studies of the interaction between *I. fumosorosea* and *P. xylostella* at the post-transcriptional level.

## Ethics statement

Our work confirms to the legal requirements of the country in which it was carried out.

## Author contributions

Conceived and designed the experiments: FJ, MS, and XiaoxX. Performed the experiments: JX and XiaoxX. Analyzed the data: MS, XiaojX, JY, XZ, and JX. Contributed reagents, materials, analysis tools: SL and SW. Wrote the manuscript: MS, JX, and XiaoxX. Revised the manuscript: MS, FJ, and XY.

### Conflict of interest statement

The authors declare that the research was conducted in the absence of any commercial or financial relationships that could be construed as a potential conflict of interest.
