# Supplementary figures and images for: Genome-Wide Profiling of Plutella xylostella Immunity-Related miRNAs after Isaria fumosorosea Infection
Source: Front Physiol. 2017 Dec 14;8:1054. doi: 10.3389/fphys.2017.01054 (PMC5735356; doi:10.3389/fphys.2017.01054)

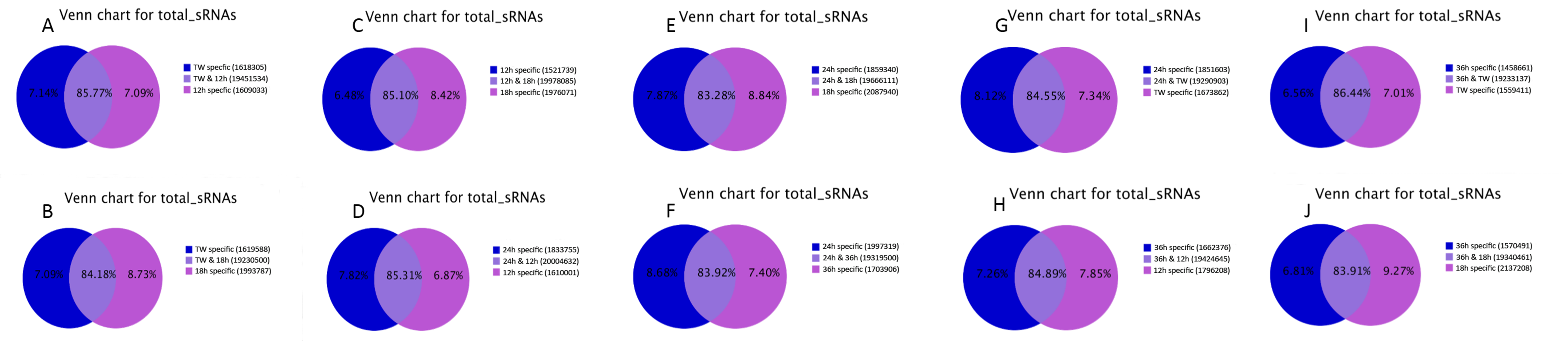

Supplement: Supplementary file 1 [file Image1.TIF]

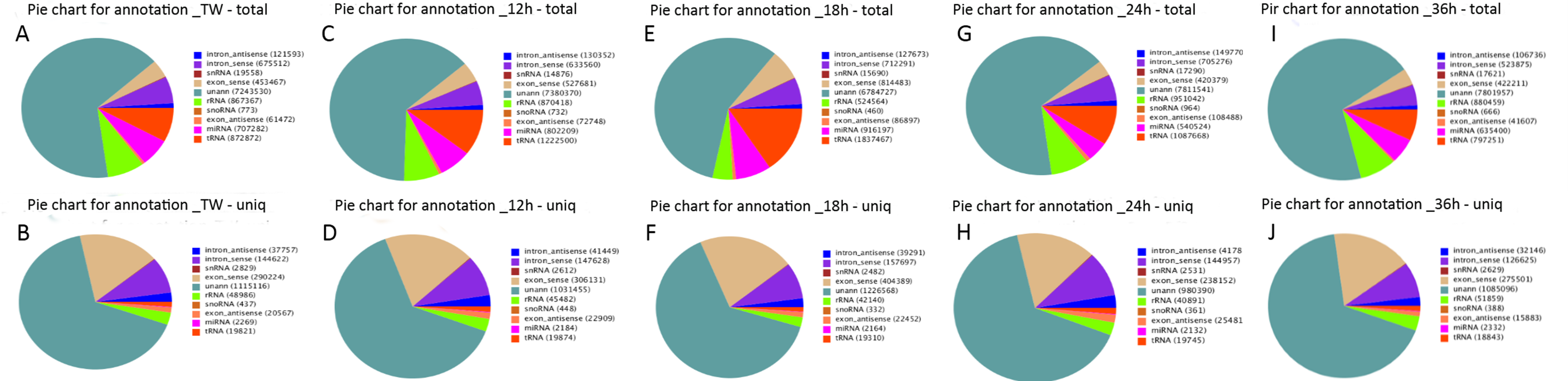

Supplement: Supplementary file 2 [file Image2.TIF]

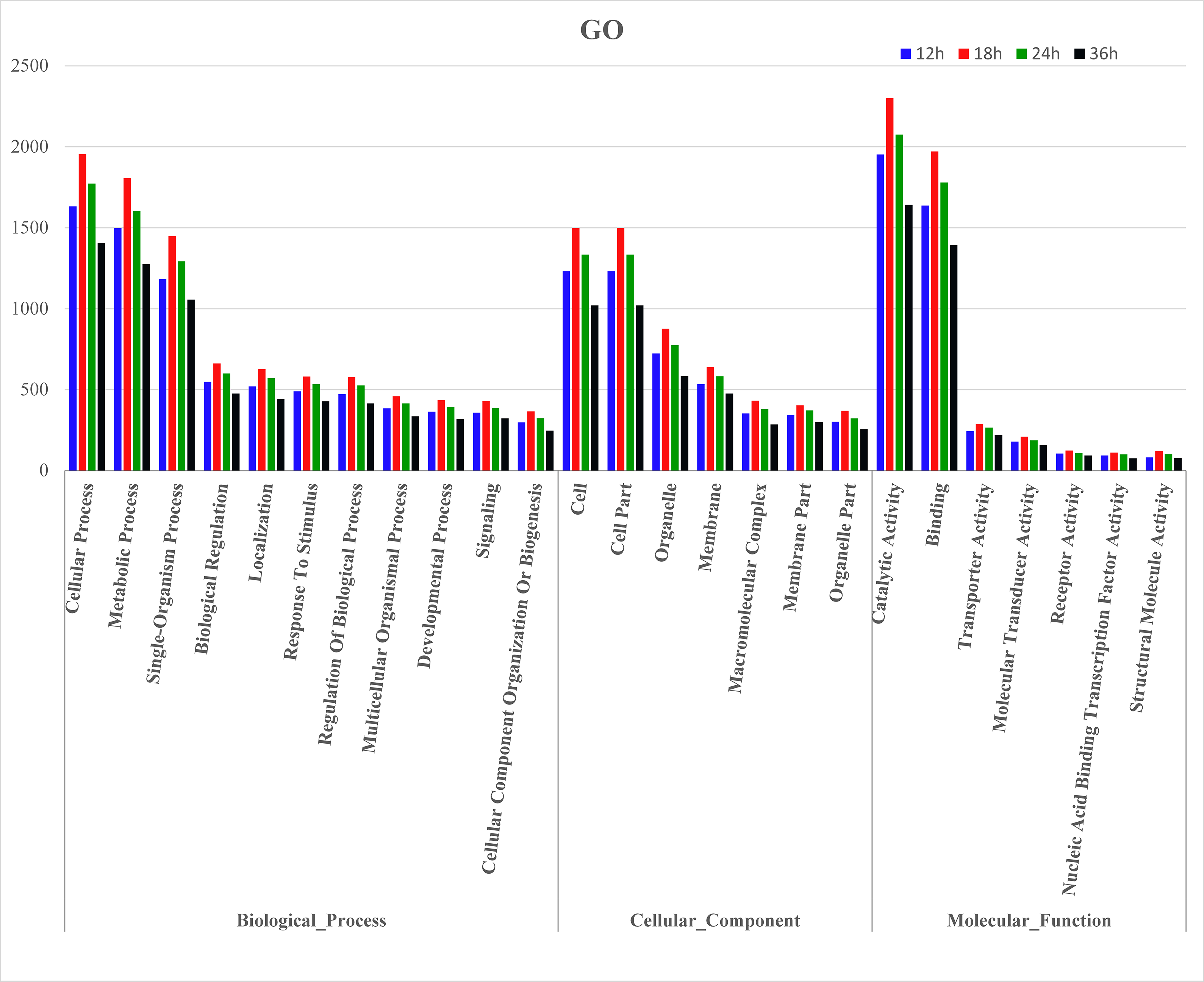

Supplement: Supplementary file 3 [file Image3.TIF]

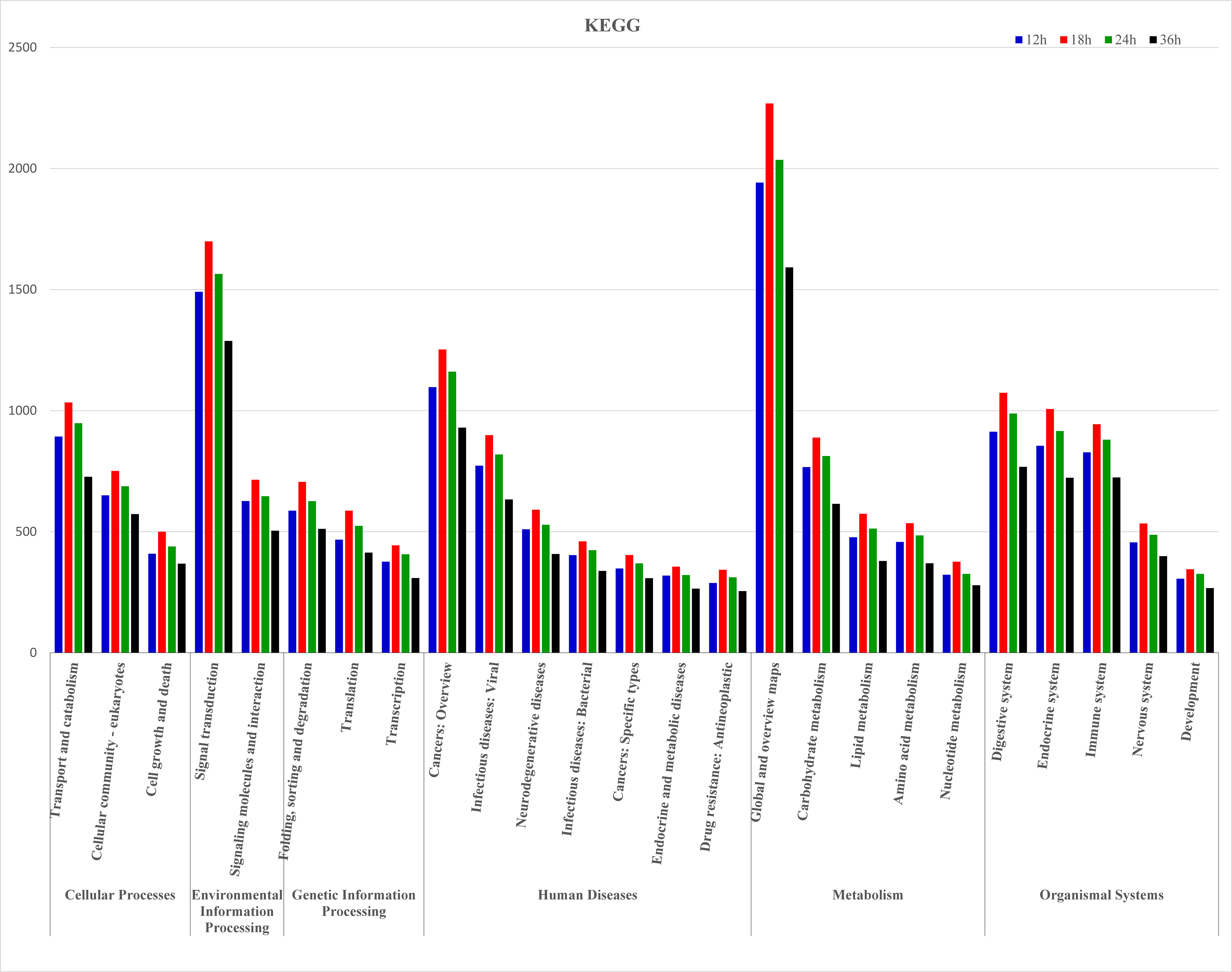

Supplement: Supplementary file 4 [file Image4.TIF]
