# Supplementary material for: Genome-Wide Profiling of Plutella xylostella Immunity-Related miRNAs after Isaria fumosorosea Infection
Source: Front Physiol. 2017 Dec 14;8:1054. doi: 10.3389/fphys.2017.01054 (PMC5735356; doi:10.3389/fphys.2017.01054)
Supplement: Supplementary file 11 [file DataSheet1.DOCX]

**Supplementary information Figure 1. The common small RNAs (sRNAs) of *Plutella xylostella* at different time points.**

summary of total sRNAs between control and 12h (B) summary of total sRNAs between control and 18h (C) summary of total sRNAs between 12h and 18h (D) summary of total sRNAs between 24h and 12h (E) summary of total sRNAs between 24h and 18h (F) summary of total sRNAs between 24h and 36h (G) summary of total sRNAs between 24h and control (H) summary of total sRNAs between 36h and 12h (I) summary of total sRNAs between 36h and control (J) summary of total sRNAs between 36h and 18h

Note; TW (Tween) was used as a control.

**Supplementary information Figure 2. Small RNA composition of Control, 12h, 18h, 24h and 36h in *P. xylostella***

Note; TW (Tween) was used as a control.

**Supplementary information Figure 3. Gene ontology functional classification of microRNA target genes at different time points post-infection**

The horizontal coordinates are class of GO, left vertical coordinates are percentage of microRNA target genes, and the right vertical coordinates are number of microRNA target genes

**Supplementary information Figure 4.** **KEGG pathway annotation classification of microRNA target genes at different time points post-infection**

The abscissa is the KEGG classification, and the ordinate left is the gene number.

**Supplementary information Table 1.** The mapping statistics of sRNAs from five libraries of *Plutella xylostella*

**Supplementary information Table 2.** Known miRNAs with their precursor sequences in the five libraries of *Plutella xylostella*

**Supplementary information Table 3.** Known miRNAs without precursor sequences in the five libraries of *Plutella xylostella*

**Supplementary information Table 4.** Novel miRNAs with their precursor sequences in the five libraries of *Plutella xylostella*

**Supplementary information Table 5.** Different expression of known miRNAs between control and 12h, 18h, 24h, and 36h in *Plutella xylostella*

**Supplementary information Table 6.** Different expression of novel miRNAs between control and 12h, 18h, 24h, and 36h in *Plutella xylostella*
